# Visual Acuity Examination Methodology in Keratoconus

**DOI:** 10.3390/jcm12247620

**Published:** 2023-12-11

**Authors:** Magdalena Nandzik, Edward Wylęgała, Adam Wylęgała, Dominika Szkodny, Anna Maria Roszkowska, Ewa Wróblewska-Czajka

**Affiliations:** 1 Department of Ophthalmology, District Railway Hospital in Katowice, Medical University of Silesia, 40-760 Katowice, Poland; 2 Ophthalmology Clinic, Department of Biomedical Sciences, University of Messina, 98100 Messina, Italy

**Keywords:** keratoconus, logMAR conversion, visual acuity, Snellen chart, ETDRS chart, logMAR conversion

## Abstract

Visual acuity is one of the most important parameters for evaluating the vision of patients with keratoconus. This study reviewed 295 articles related to keratoconus published between 2017 and 2022 in which visual acuity was one of the parameters measured. The methodology of visual acuity testing in studies on keratoconus was thoroughly analyzed. The analysis showed that the most commonly indicated chart for testing visual acuity papers on keratoconus is the Snellen chart. It was shown that in 150 out of 295 articles, the authors do not describe the methodology for testing visual acuity. What is more, it was also shown that in 68 of the 295 articles which were analyzed, a procedure for converting visual acuity tested with a Snellen chart into a logMAR scale was used. In this review, we discuss the validity and reliability of such conversions. In particular, we show that insufficient description of visual acuity testing methodology and lack of information on the conversion of visual acuity results into the logMAR scale may contribute to the misinterpretation of visual acuity test results.

## 1. Introduction

Visual acuity testing is the most elementary part of ophthalmic examinations. The purpose of this test is to assess the resolving power of the central part of the retina, known as the macula, which is also influenced by the resolving power of the optical system in front of the retina. Visual acuity testing assesses the ability to perceive two objects close together as separate objects [1]. Visual acuity commonly refers to the clarity of vision, but technically rates a person’s ability to recognize small details with appropriate precision. Optotype charts are used to test visual acuity. The most commonly used are the Snellen charts and the ETDRS charts [2]. Less commonly used optotype charts include Landolt C, Lea Symbols, Tumbling E, and Allen [3,4]. The Snellen chart was first described and used in 1862 by famous Dutch doctor of medicine and ophthalmologist Herman Snellen. The chart has optotypes of different sizes arranged from largest to smallest, which are read with each eye separately from a specific distance, usually 5 or 6 m [5]. The Snellen chart has several disadvantages, such as variable letter size, a full-line test method, irregular progression between lines, and uneven character legibility [6]. Hence, these factors contribute to the poor repeatability of visual acuity measurement [7]. We need to be aware that the Snellen charts that are in use are not standardized and may have different fonts, letters, and spacing ratios. What is more, they may also be displayed in different ways and have different background brightness. Currently, the ETDRS chart is considered the ‘gold standard’ for testing visual acuity in clinical trials [8]. It was developed from the Bailey–Lovie chart [9]. Its advantages are equal legibility of letters, consistent letter and line spacing, and equal logarithmic letter size spacing between lines [10]. Each letter read is assigned a value of 0.02 logMAR [11]. When using the logMAR table or other non-standardized logarithmic tables, visual acuity is assessed based on the logarithm of the minimum angle of resolution. What is important, an observer who can resolve details as small as 1 min of visual angle has a logMAR acuity of 0 because the logarithm of the base 10 of 1 is 0. In contrast to the Snellen chart, the ETDRS chart is standardized, making it a recommended tool for scientific research. The disadvantages of using ETDRS charts are longer testing time, insufficient knowledge of testing protocols, the need for staff training, and high price [12].

Keratoconus is a bilateral and usually asymmetric disease [13] which results in progressive thinning and steeping of the cornea leading to irregular astigmatism and decreased visual acuity [14]. Traditionally, the condition has been described as a non-inflammatory disease. However, more recently, it has been associated with ocular inflammation. Keratoconus usually develops in the second and third decades of life and progresses into the fourth decade of life. What is more, progressive stromal thinning, rupture of the anterior limiting membrane, and subsequent ectasia of the central or paracentral cornea are the most common changes in changing the structure of the cornea. The disease occurs in all ethnicities and all genders. Risk factors for developing keratoconus are a family history of keratoconus, eye rubbing, eczema, asthma, and allergy [15,16,17]. The prevalence and incidence rates of keratoconus have been estimated to be between 0.2 and 4790 per 100,000 persons and 1.5 and 25 cases per 100,000 persons/year, respectively, with highest rates typically occurring in 20- to 30-year-olds and in Middle Eastern and Asian ethnicities [18,19].

According to our observations, patients with keratoconus often confuse single letters when checking visual acuity (this is most likely related to the occurrence of irregular astigmatism). It would seem that the most advantageous method of testing visual acuity, due to taking into account the number of letters read, would be to use the ETDRS chart. It was decided to look at how visual acuity in patients with keratoconus is tested in scientific research. This is the reason why we decided to investigate the methodology of visual acuity testing in patients with keratoconus.

This paper reviews publications related to the topic of keratoconus, published between 2017 and 2022, in which visual acuity was one of the parameters measured. The main aim of this paper is to review the literature on visual acuity testing in patients with keratoconus and, in particular, to look at visual acuity testing methodology, notation of results, and their presentation.

## 2. Materials and Methods

**Figure 1:**
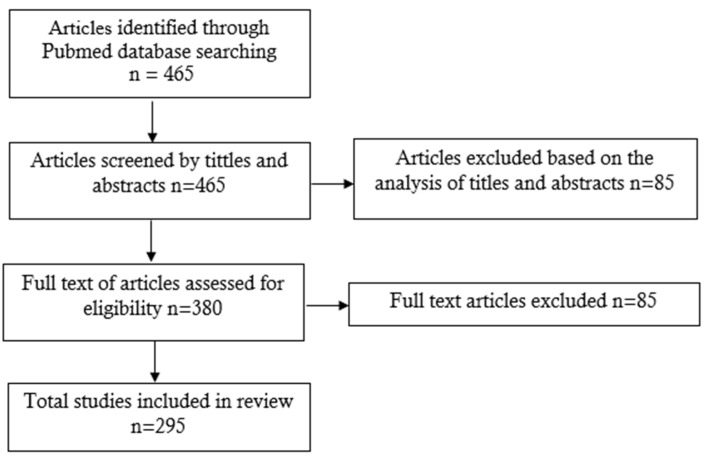
Study flow diagram.

The keyword “Keratoconus visual acuity” was searched in the PubMed database, and the search area was narrowed to full-text articles in English which were published between 2017 and 2022. The search returned 465 results. After analyzing the titles and abstracts, 85 papers were rejected. These included meta-analyses and case studies. After that, based on the analysis of the full texts, eligibility evaluation was performed. After evaluation, 85 papers were eliminated. Finally, a total of 295 articles were eligible for analysis. Below we present the figure (Figure 1) which shows the a study flow diagram.

The analysis of each of the 295 publications consisted, first of all, in looking for information in the part of the work devoted to materials and methods. If the information on the type of visual acuity test could not be found in this section of the article, we still made an attempt to search for information through searching for specific phrases in the text, namely “visual acuity”, “Snellen”, “chart”, “logMAR”, “pachymetry”, “keratometry”, “decimal scale”, and “logarithmic scale”.

## 3. Results

### 3.1. Analysis of Visual Acuity Testing Methodology

A total of 295 articles fulfilled the criteria for being included in the main analysis to determine how visual acuity is tested in patients with keratoconus. The obtained results are presented in Table 1. The final analysis showed that in 35 articles, the visual acuity test was performed using the ETDRS chart. Then, in 10 articles, it was indicated that the examination was performed using other charts, i.e., Tumbling E (2), Landolt C (2), Bailey-Lovie (1), and Sloan (1). Next, in two cases, testing was performed using COMPlog computerized visual acuity testing systems, Medisoft Inc., Leeds, UK. After that, in 103 articles, visual acuity testing was performed using Snellen charts. Finally, in 150 articles, no information was given on which charts were used to perform the visual acuity test. Only in one publication were both the logMAR and Snellen charts used.

### 3.2. Analysis of Visual Acuity Results Presentation Methodology in Articles

A total of 295 articles were analyzed for how visual acuity results are presented. The obtained results are presented in Table 2. The analysis showed that in 215 articles, results were presented using the logMAR scale, while in 62 articles, the results were analyzed as a fraction or decimal scale. In seven articles, authors analyzed increments of rows of letters or the number of letters read. In 11 papers, results were reported both using the logMAR scale and as a fraction or decimal scale.

### 3.3. Conversion of Visual Acuity Values Examined Using Snellen Charts to LogMAR in Articles

Of the 295 articles reviewed, there were 68 authors who acknowledged that visual acuity was measured with Snellen charts converted to the logMAR scale. In 106 articles reviewed, the method of testing visual acuity is not stated, but the result was presented on the logMAR scale. This raises the question of whether the work used the ETDRS chart or whether the result was recalculated. Table 3 shows quantitative summary of articles in which visual acuity was tested using Snellen chart and converted to the logMAR scale, relative to other analyzed articles.

### 3.4. Actual Scale of Practice of Converting Visual Acuity Measured with Snellen Charts to LogMAR Scale

As already mentioned in 106 of the reviewed papers, the method of testing visual acuity was not stated, but the final result was presented on the logMAR scale. This can be interpreted in two ways. It can be assumed that the test was carried out using logarithmic charts or that the results were converted to the logMAR scale. Therefore, it must be concluded that the number of papers in which visual acuity values were converted may be higher. Table 4 shows quantitative summary of articles in which visual acuity was recalculated versus those in which a logMAR score was reported but the study methodology and other publications were not reported.

Table 5 presents a summary of 25 of the 295 analyzed articles whose titles contained the phrases “visual acuity”, “refractive error”, and “visual results”. It is worth noting that even in publications which were strictly related to visual acuity testing, the methodology of visual acuity testing varied and there were deficiencies in reporting the details of acuity testing. A table was also created to show the study database prepared for the purposes of this publication.

## 4. Discussion

Of the 295 papers, 145 (49.2%) indicated the visual acuity method used. The most frequently used chart was the Snellen chart: it was used in 103 cases, which constitutes 34.9% of the reviewed papers. In second place was the ETDRS table, used in 35 papers, which constitutes 11.8% of the analyzed articles. A total of 150 papers, which constitutes 50.8% of all papers, did not provide the exact optotype table used for the study.

For comparison, the only work found on a topic similar to ours, authored by Williams, M.A et al. [44], showed that out of 128 analyzed works, 58.6% indicated the type of table used to test the visual acuity. This result is 9.4% higher than that obtained in our work. The authors of this work also showed that the most frequently used table was the ET-DRS chart: it was used in 34 analyzed articles, which constituted 26.6% of all papers. The second most frequently used chart was the Snellen chart, which was used in 32 papers, constituting 25% of the articles. The authors of this work showed that thirty-six articles, 28.1% of all analyzed papers, did not specify the type of optotype chart used for visual acuity testing. As you can see, the percentage of analyzed papers in which authors do not describe the visual acuity testing methodology used is lower than in our study. This may be due to th fact that the authors of the mentioned article analyzed works published in five ophthalmology journals belonging to the leading clinical ophthalmology journals, with high requirements for the presentation of acuity test results and high impact factors. The use of logMAR ETDRS charts has been considered the gold standard in visual acuity testing because it provides more accurate results compared to other visual acuity charts and the results are easier to use in vision analysis, making it the preferred chart in clinical trials [45]. Even so, we showed that the ETDRS table was only used in 35 papers, which does not seem to be a very large number.

It was shown that of the 295 publications analyzed, 68 used a procedure to convert visual acuity tested with a Snellen-type chart to the logMAR scale. It is worth noting that in 150 of the articles analyzed, the method of testing visual acuity was not explicitly specified, of which 106 reported the result on the logMAR scale. This state of affairs can be interpreted in two ways. It can be assumed that the test was carried out using logarithmic charts or the results were converted to the logMAR scale. Therefore, it must be takn into account that the number of papers in which visual acuity values were converted may be higher.

For the purposes of this discussion, in order to be able to comment on the appropriateness of using conversions, it was decided to review publications examining differences in visual acuity obtained using Snellen and ETDRS charts.

A paper by Hannah et al. [46] compared best corrected visual acuity in people with AMD examined using the Snellen chart and the ETDRS chart. The study included 413 people. Before comparison, the score obtained using the Snellen chart was converted to the logMAR scale and compared with the results obtained using the ETDRS chart. Visual acuity scores were significantly better using the ETDRS charts compared with the Snellen score converted to the logMAR scale. The differences were greatest in those with poor visual acuity. Researchers Kaiser [47] and Falkenstein et al. [48] reached similar conclusions in their publications. In both papers, researchers compared visual acuity tested with ETDRS and Snellen charts. Kaiser’s study was conducted on 163 patients with a variety of ophthalmic conditions. Falkenstein et al. compared visual acuity tested with the ETDRS and the Snellen charts in 190 eyes. Before the comparison was prepared, the score obtained using the Snellen chart was converted to the logMAR scale and compared with the results obtained using the ETDRS chart. In both studies, visual acuity scores were significantly better using the ETDRS charts compared with the Snellen score converted to the logMAR scale. The differences were greatest in those with poor visual acuity (<20/200). Interestingly, similar conclusions were reached by Baker et al. [49]. In the conclusion of the paper, it can be found that corrected visual acuity results obtained using the ETDRS chart resulted in significantly better results compared to Snellen VA measurements. The differences were more pronounced for eyes with reduced visual acuity. A different conclusion was reached by Kalpana and Jayaraji [50], who compared best corrected visual acuity tested with the Snellen chart, which was then converted to the logMAR scale, with results obtained using the ETDRS chart. The study was conducted in up to 630 eyes. After analyzing the data, the authors concluded that there were no statistically significant differences between the parameters and the charts could therefore be used interchangeably. What is particularly noteworthy is that the average visual acuity in the study group was 0.043 logMAR. This means that people with very good visual acuity were studied. Unfortunately, it was not possible to find a study in which visual acuity tested with Snellen-type and ETDRS charts in patients with keratoconus was compared.

It seems that the conversion of visual acuity values tested using Snellen charts to the logMAR scale should not take place. This is an action that may indicate a misunderstanding of the principle of individual visual acuity charts. Recalculation leads to the falsification of the visual acuity results obtained.

Elliott [51] even goes so far as to claim that the conversion of visual acuity results obtained using Snellen charts to the logMAR scale may cause an artificial increase in the reliability of the tests performed.

In one of the reviewed articles, by Rakhshandadi T et al. [52], it was argued that visual acuity values are converted to logMAR for statistical analysis. With results obtained using Snellen charts, during statistical analysis one may encounter difficulty in obtaining normality of distribution. For this reason, logarithmizing the data is a common statistical procedure, and it is completely correct. However, the statistical analysis section of the paper should then state that the data have been logarithmized.

Mirataollah S et al. [53], whose conclusions compared visual acuity measured using Snellen charts and ETDRS charts, indicate that there are differences between the results obtained. They point out that particular caution should be exercised when interpreting and comparing visual acuity results in retrospective studies in which Snellen charts are more often used compared to clinical studies in which ETDRS charts are more often used.

The second important point that we would like to discuss is that in 150 out of 295 analyzed publications, the authors did not indicate which method of visual acuity was tested. It seems that this number is large, and more attention should be paid to describing the methodology of the visual acuity tests performed. All data on the methodology of visual acuity testing should be described in a clear, comprehensive manner in the section of the text that is devoted to this topic, i.e., materials and methods.

In addition, more attention should be paid to the quality of presentation of the test results conducted, including visual acuity testing methods. The choice of test chart is important, too. It is the responsibility of the researchers to reliably and honestly report on the methodology of the tests performed.

To summarize the discussion, converting tested visual acuity obtained using the Snellen chart to logMAR is a questionable practice that may falsify the results obtained. In addition, presenting converted results without explicitly stating this in the text of the publication is a questionably fair practice. It seems that the conversion of visual acuity examined using the Snellen chart in patients with keratoconus into the ETDR scale should not take place. To state this conclusively, it would be necessary to conduct new research on this topic, which has not been studied yet.

Visual acuity converted to logMAR should not be compared with that measured on the ETDRS chart. The methodology of visual acuity testing should be carefully analyzed when researchers intend to compare obtained visual acuity results with those of other studies or are working on meta-analyses, for example. That is why it is so important to accurately describe the methodology of visual acuity testing and inform about the conversion of visual acuity results to the logMAR scale.

## 5. Conclusions

Visual acuity testing in patients with keratoconus is performed in a very diverse manner. The reason for this state of affairs is certainly the lack of standards regarding the quality of visual acuity testing in scientific research, including that related to keratoconus.

Researchers rarely use standardized visual acuity tests, even though they are undoubtedly the most appropriate method of monitoring visual acuity in scientific research.

It is obvious that researchers are obliged to provide reliable and honest information about the methodology of the conducted research, including on visual acuity. All data regarding visual acuity testing methodologies should be described in a clear and comprehensive manner. This study showed that researchers should pay more attention to the methodology and method of presenting visual acuity test results.

We would like to propose four principles for collecting and analyzing visual acuity data in patients with keratoconus:

1. If possible, use standardized visual acuity charts;
2. If you do not use standardized tables to test visual acuity, try to describe the test methodology well. Specify the type of table used (logarithmic or decimal). Provide the manufacturer of the optotype table, etc.;
3. If you care about the quality of your examinations, try not to convert visual acuity values tested using Snellen charts into the logMAR scale and vice versa, unless it is necessary;
4. If you convert visual acuity values, e.g., from the Snellen fraction to the logMAR scale and vice versa, please inform about it in the text of the article.

The principles created are universal, and we believe that it is worth following them in all research related to visual acuity testing. These activities will help improve the quality of scientific and research work related to keratoconus, but also research on other ophthalmological diseases.

## Figures and Tables

**Table 1:** Number of papers in which each visual acuity test method was used.

Type of Chart	Number of Papers
Snellen	103
ETDRS	35
Tumbling E	2
Landolt C	2
Bailey–Lovie	1
Sloan	1
COMPlog, Medisoft Inc.	2
Not specified	150

**Table 2:** Number of articles in which different methods of presenting visual acuity results were used.

Methods of Presenting the Results of Visual Acuity Testing	Number of Papers
LogMAR scale	215
Fraction or decimal scale	62
Increments of letters or rows of letters	7
LogMAR scale and fraction or decimal scale	11

**Table 3:** Quantitative summary of articles in which visual acuity was tested using Snellen chart and converted to the logMAR scale, relative to other analyzed articles.

Number of Papers in Which Acuity Tested with Snellen Chart Was Converted to LogMAR Scale	Number of Other Papers
68	227

**Table 4:** Quantitative summary of articles in which visual acuity was recalculated versus those in which a logMAR score was reported but the study methodology and other publications were not reported.

	Number of Papers
Publications in which visual acuity was tested using the Snellen chart and was converted to logMAR	68
Publications in which the testing methodology was not stated but the result was presented on the logMAR scale (converted or tested using ETDRS?)	106
Other publications	121

**Table 5:** List of 25 out of 295 analyzed articles, whose titles include the phrases “visual acuity”, “refractive refractions”, “refractive error”, and “vision results”.

Article	Design	Number of Participants	Chart Used, Results
Aneeq Ansari et al. [20]	retrospective	65 eyes of 65 patients	Snellen chart, results in fraction notation
Muijzer et al. [21]	prospective	100 eyes of 50 patients	ETDRS chart, results in logMAR scale
Grišević et al. [22]	prospective	44 eyes of 34 patients	Snellen chart, results in decimal notation
Amanzadeh et al. [23]	prospective	42 eyes of 32 patients	visual acuity test method not indicated, results in logMAR scale
Hassani et al. [24]	prospective	28 eyes	visual acuity test method not indicated, results in logMAR scale
Liduma et al. [25]	prospective	77 eyes of 44 patients	visual acuity test method not indicated, results in decimal notation
Zarei-Ghanavati et al. [26]	prospective	22 eyes of 11 patients	ETDRS chart, results in logMAR scale
Arej et al. [27]	retrospective	31 eyes	ETDRS chart, results in logMAR scale
Iqbal et al. [28]	retrospective	28 patients	visual acuity test method not indicated, results in logMAR scale
Godeefrooij et al. [29]	prospective	61 patients	visual acuity test method not indicated, results in logMAR scale
Jabbarvand et al. [30]	retrospective	34 patients	Snellen chart, visual acuity converted to logMAR, results in logMAR scale
Csorba et al. [31]	retrospective	47 eyes of 47 patients	Snellen chart, visual acuity converted to logMAR, results in logMAR scale
Amer et al. [32]	prospective	68 eyes of 35 pediatric patients	Snellen chart, results in decimal notation
Yüksel et al. [33]	prospective	76 eyes of 71 patients	visual acuity test method not indicated, results in logMAR scale
Heikal et al. [34]	retrospective	30 eyes of 20 patients	visual acuity test method not indicated, results in logMAR scale
El-Khoury et al. [35]	retrospective	26 eyes of patients ≤ 18 years, and 26 eyes of adult patients as controls.	visual acuity test method not indicated, results in logMAR scale
Tognon et al. [36]	retrospective	1222 eyes of 1196 patients	Snellen chart, visual acuity converted to logMAR, results in logMAR scale
Janiszewska-Bil et al. [37]	prospective	90 eyes of 90 patients	Snellen chart, results in decimal notation and logMAR scale
Stevenson et al. [38]	retrospective	71 eyes of 71 patients	Snellen chart, visual acuity converted to logMAR, results in logMAR scale
Krysik et al. [39]	retrospective	42 eyes of 34 patients	Snellen chart, results in decimal notation
Buzzonetti et al. [40]	retrospective	20 eyes of 20 patient	visual acuity test method not indicated, results in logMAR scale
Bozkurt et al. [41]	retrospective	47 eyes of 41 patients	ETDRS chart, results in logMAR scale
Hashemian et al. [42]	prospective	71 eyes of 52 patients	visual acuity test method not indicated, results in logMAR scale and fraction notation
Nicula et al. [43]	retrospective	61 eyes of 35 patients	Snellen chart, results in logMAR scale

## Data Availability

Data are available on request due to restrictions (e.g., privacy or ethics).
